# Assessment of Ethno-racial and Insurance-based Disparities in Pediatric Forearm and Tibial Fracture Care in the United States

**DOI:** 10.5435/JAAOSGlobal-D-22-00126

**Published:** 2022-07-29

**Authors:** Andrew J. Landau, Afolayan K. Oladeji, Pooya Hosseinzadeh

**Affiliations:** From the Department of Orthopaedic Surgery; Washington University School of Medicine, St. Louis, MO.

## Abstract

**Introduction:**

Despite growing attention to healthcare disparities and interventions to improve inequalities, additional identification of disparities is needed, particularly in the pediatric population. We used state and nationwide databases to identify factors associated with the surgical treatment of pediatric forearm and tibial fractures.

**Methods:**

The Healthcare Cost and Utilization Project State Inpatient, Emergency Department, and Ambulatory Surgery and Services Databases from four US states and the Nationwide Emergency Department Sample database were quarried using International Classification of Diseases codes to identify patients from 2006 to 2015. Multivariable regression models were used to determine factors associated with surgical treatment.

**Results:**

State databases identified 130,006 forearm (1575 open) and 51,979 tibial fractures (1339 open). Surgical treatment was done in 2.6% of closed and 37.5% of open forearm fractures and 7.9% of closed and 60.5% of open tibial fractures. A national estimated total of 3,312,807 closed and 46,569 open forearm fractures were included, 59,024 (1.8%) of which were treated surgically. A total of 719,374 closed and 26,144 open tibial fractures were identified; 52,506 (7.0%) were treated surgically. Multivariable regression revealed that race and/or insurance status were independent predictors for the lower likelihood of surgery in 3 of 4 groups: Black patients were 43% and 35% less likely to have surgery after closed and open forearm fractures, respectively, and patients with Medicaid were less often treated surgically for open tibial fractures in state (17%) and nationwide (20%) databases.

**Conclusions:**

Disparities in pediatric forearm and tibial fracture care persist, especially for Black patients and those with Medicaid; identification of influencing factors and interventions to address them are important in improving equality and value of care.

Racial and socioeconomic barriers to equal quality health care have been reported in recent decades, including orthopaedic^1,2,3,4,5,6,7,8,9,10,11,12,13,14,15^ and nonorthopaedic^16-24^ conditions in adult and pediatric populations. These disparities may have notable clinical, patient satisfaction and economic consequences, with healthcare inequalities totaling over $300 billion in excess healthcare costs, including direct medical payments and indirect costs related to factors such as lost productivity because of preventable illnesses and complications.^17,25,26^ Despite efforts to mitigate these factors, barriers persist regarding access, quality, and treatment. Long bone fractures are common in the pediatric population and require substantial healthcare resources to ensure appropriate treatment and avoid long-term complications. Therefore, identifying barriers to care and using interventions to improve treatment equality are important for both medical professionals and policymakers.

Previous studies have used population-based epidemiological data to identify discordant treatment of some pediatric long bone fractures, including closed supracondylar humerus, femoral shaft, forearm, and other extremity fractures.^8,10,13^ Studies have also demonstrated discrepancies to equal access based on insurance status,^4,10,11^ race and/or socioeconomic disparities in evaluation and treatment,^3,7,8,13^ access to timely management,^4,6^ follow-up care,^5,11,12,14^ and readmission rates/repeat ED visits, among others.^4,5,7,11,12,14,20,21^

Although some have attempted to investigate the relationship between race/ethnicity and sociodemographic status and pediatric forearm and tibial fracture treatment, data are inadequate and additional investigation is needed to establish a true estimate of the prevalence and effect of these factors on treatment. We sought to expand on the current literature by evaluating these relationships in the most common upper and lower extremity pediatric fracture types, using large multistate and national databases to better identify these factors and inform strategies to understand and address barriers to care. We hypothesized that ethno-racial and insurance-based disparities affect the type of treatment (surgical versus nonsurgical) received in pediatric forearm and tibial fractures.

## Materials and Methods

### Data Sources

The Healthcare Cost and Utilization Project (HCUP) State Inpatient Databases, State Emergency Department Databases, and State Ambulatory Surgery and Services Databases, sponsored by the Agency for Healthcare Research and Quality, from Maryland, New York, Vermont, and Wisconsin prospectively collect statewide longitudinal information on inpatient discharges from nonfederal hospitals, emergency department discharges, and encounter-level ambulatory and outpatient services data, respectively.^27-29^ These four states were chosen because they provided the largest data sets available. They allow for comparison between these states and national data to help identify geographic disparities or confounding variables at the state level. These databases provide clinical and nonclinical variables such as demographic information including race and ethnicity, diagnosis, and procedure codes for multiple payer types; however, they do not include data for uninsured patients. Capture of longitudinal data allows for the assessment of subsequent encounters.

The Nationwide Emergency Department Sample (NEDS) database from the HCUP/AHQR provides similar clinical and nonclinical data to the state databases (except ethno-racial data) and more detailed injury information such as mechanism, associated charges, and visit data from hospital-owned emergency department discharges.^30^ The NEDS also provides a 20% stratified sample of nationwide data that can be used to produce weighted national estimates.

Both databases are based on paid insurance claims (Medicaid and private insurance). These databases have been used for prior investigations of orthopaedic and nonorthopaedic conditions^3,4,13,22,23,24,25,26,31^ and validated for total knee arthroplasty, hip fracture, and other orthopaedic diagnoses.^33-35^

### Population Selection

We used International Classification of Diseases, Ninth Revision, Clinical Modification codes to identify four groups of patients (Appendix, http://links.lww.com/JG9/A228): closed forearm fractures: 129,431 records from state databases and 724,305 true records (weighted national estimate: 3,312,807) from NEDS; open forearm fractures: 1575 state records and 10,035 (weighted estimate: 46,569) from NEDS; closed tibial fractures: 50,640 state records and 223,524 (weighted estimate: 719,374) from NEDS; and open tibial fractures: 1339 state records and 6215 (weighted estimate: 26,144) from NEDS. Patients aged 1 to 18 years were included, and all records with corresponding diagnosis codes were assessed.

### Predictors

For each diagnosis, patient age, sex, race, insurance status, mechanism of injury, fracture type (open versus closed), treatment (surgical versus nonsurgical), facility type, and location were extracted from the appropriate databases and analyzed as predictors of care. Racial/ethnic groups were available only from state databases and defined as White, Black, Hispanic, and other/missing. Insurance status was characterized as Medicaid/government-assisted and all other payer types.

### Outcome Measures

The primary outcome was the treatment method for each fracture type. Groups were subdivided into surgical versus nonsurgical groups based on International Classification of Diseases, Ninth Revision, Clinical Modification and Current Procedural Terminology treatment codes (Appendix, http://links.lww.com/JG9/A228). Groups for each diagnosis were assessed to determine the effect of sex, age, race/ethnicity (state databases), and payer type on the treatment received. Complications such as compartment syndrome, infection, readmission, and surgery within 90 days were recorded.

### Statistical Analysis

State databases: Demographic data were described using quantity and proportion of the whole and of each fracture group. Patients were characterized by sex, age, race/ethnicity, and insurance status. The chi square or Fisher exact test was used for categorical variables and the Kruskal-Wallis test for continuous variables. Multivariable generalized estimating equation models were used to evaluate the effect of these traits on treatment type among each subgroup while controlling for all other patient and injury characteristics. Relative risks (RR) were calculated to estimate the likelihood of surgical treatment related to each variable. All eligible variables were included in the models.

NEDS database: Demographic data and proportions were reported as described earlier, along with national weighted estimates of frequencies with standard errors, which estimate the prevalence of each variable in the United States meeting identical inclusion criteria. For example, we identified 724,305 true records for closed forearm fractures; however, the weighted national estimate was 3,312,807 cases in the United States for the study period. Multivariable regression models were used to assess factors predictive of the index treatment method (surgical versus nonsurgical). Specifically, the effect of age, sex, and primary payer type were analyzed, and odds ratios (OR) were used to determine the odds of surgical treatment for each variable.

For both databases, regression models were used separately for open and closed fractures because clinical rates of surgical management were too different between open and closed fractures to accurately compare them as a single population (ie, all forearm or all tibial fractures). Analyses were conducted using SAS software (version 9.4-SAS Institute).

## Results

A total of 1,143,150 records for pediatric forearm (864,346) and tibial (281,718) fractures were included from state (130,006 forearm, 51,979 tibia) and national (734,340 forearm, 229,739 tibia) databases from 2006 to 2015 (MD 2013 to 2015, NY 2006 to 2015, VT 2011 to 2015, WI 2013 to 2015, NEDS 2006 to 2015). The mean age (standard deviation) was 10.0 ± 4.5 years for patients from state-based data. Most patients were male (64%); the most common race/ethnicity was White (62%). Patients from the NEDS were categorized into two age groups: preteen (age 1 to 12.9 years) and teen (13 to 18 years), representing 70.4% and 29.6% of the population, respectively. Demographic information is presented in Tables 1 and 2.

**Table 1:** Demographics and Database Results for Forearm Fractures

Forearm Fractures				
State Databases		Closed Fracture	Open Fracture	Total
Number of cases	N (%)	128,431 (98.8)	1575 (1.2)	130,006
Age (years)	Mean (SD)	9.7 (4.2)	11.1 (4.2)	9.7 (4.2)
		N (%)		
Sex	Female	46,654 (36.3)	577 (36.6)	47,231 (36.3)
	Male	81,777 (63.7)	998 (63.4)	82,775 (63.7)
Race/Ethnicity	White	82,529 (64.3)	913 (58.0)	83,442 (64.2)
	Black	14,804 (11.5)	234 (14.9)	15,038 (11.6)
	Hispanic	14,773 (11.5)	203 (12.9)	14,976 (11.5)
	Other	16,325 (12.7)	225 (14.3)	16,550 (12.7)
Insurance	Medicaid	32,837 (25.6)	481 (30.5)	33,318 (25.6)
	Other payer	95,594 (74.4)	1094 (69.5)	96,688 (74.4)
Treatment type	Surgical	3386 (2.6)	591 (37.5)	3977 (3.1)
7	Nonsurgical	125,045 (97.4)	984 (62.5)	126,029 (96.9)
Database	SASD	10,000 (7.8)	169 (10.7)	10,169 (7.8)
	SEDD	113,395 (88.3)	524 (33.3)	113,919 (87.6)
	SIDD	5036 (3.9)	882 (56.0)	5918 (4.6)
NEDS		N (%)		
Number of cases		724,305 (98.6)	10,035 (1.4)	734,340
Age category	Preteen	543,254 (75)	6069 (60.5)	549,323 (74.8)
	Teen	181,051 (25)	3966 (39.5)	185,017 (25.2)
Sex	Female	265,597 (36.7)	2614 (26)	268,211 (36.5)
	Male	458,708 (63.3)	7421 (74)	466,129 (63.5)
Insurance	Medicaid	240,504 (33.2)	3220 (32.1)	243,724 (33.2)
	Other payer	483,801 (66.8)	6815 (67.9)	490,616 (66.8)
Treatment type	Surgical	8251 (1.1)	4373 (43.5)	12,624 (1.7)
	Nonsurgical	716,054 (98.9)	5662 (56.4)	721,716 (98.3)

NEDS = Nationwide Emergency Department Sample, SASD = State Ambulatory Surgery and Services Databases, SEDD, State Emergency Department Databases

**Table 2:** Demographics and Database Results for Tibia Fractures

Tibial fractures				
State Databases		Closed Fracture	Open Fracture	Total
Number of cases	N (%)	50,640 (97.4)	1339 (2.6)	51,979
Age (years)	Mean (SD)	10.7 (4.96)	13.1 (4.3)	10.8 (4.96)
		N (%)		
Sex	Female	46,654 (36.3)	577 (36.6)	47,231 (36.3)
	Male	81,777 (63.7)	998 (63.4)	82,775 (63.7)
Race/Ethnicity	White	28,754 (56.8)	593 (44.3)	29,347 (56.5)
	Black	8783 (17.3)	380 (28.4)	9163 (17.6)
	Hispanic	6772 (13.4)	186 (13.9)	6958 (13.4)
	Other	6331 (12.5)	180 (13.4)	6511 (12.5)
Insurance	Medicaid	14,084 (27.8)	282 (21.1)	14,366 (27.6)
	Other payer	36,556 (72.2)	1057 (78.9)	37,613 (72.4)
Treatment type	Surgical	4041 (8.0)	837 (62.5)	4878 (9.4)
	Nonsurgical	46,599 (92.0)	502 (37.5)	47,101 (90.6)
Database	SASD	3686 (7.3)	47 (3.5)	3733 (7.2)
	SEDD	40,557 (80.1)	250 (18.7)	40,807 (78.5)
	SIDD	6397 (12.6)	1042 (77.8)	7439 (14.3)
NEDS		N (%)		
Number of cases		223,524 (97.3)	6215 (2.7)	229,739
Age category	Preteen	127,257 (56.9)	2079 (33.5)	129,336 (56.3)
	Teen	96,267 (43.1)	4136 (66.5)	100,403 (43.7)
Sex	Female	81,326 (36.4)	1633 (26.3)	82,959 (36.1)
	Male	142,198 (63.6)	4582 (73.7)	146,780 (63.9)
Insurance	Medicaid	82,904 (37.1)	2108 (33.9)	85,012 (37.0)
	Other payer	140,620 (62.9)	4107 (66.1)	144,727 (63.0)
Treatment type	Surgical	44,705 (2.0)	3729 (60.0)	48,434 (21.1)
	Nonsurgical	178,819 (98)	2486 (40.0)	181,305 (78.9)

NEDS = Nationwide Emergency Department Sample, SASD = State Ambulatory Surgery and Services Databases, SEDD = State Emergency Department Databases

### Forearm Fractures

#### State Databases

From the four state databases, 128,431 patients with closed and 1575 with open forearm fractures were included. Surgical treatment was done in 2.6% of closed and 37.5% of open forearm fracture cases.

Multivariable regression revealed that Black patients with closed fractures were 43% less likely to be treated surgically than White patients (Table 3). Male patients had a slightly higher risk of surgery, and teenagers were more commonly treated surgically than preteens. Open fractures conferred an increased risk of surgical management (RR = 13.64, 95% confidence interval [CI]: 11.60, 16.04); however, Black patients were less likely to have surgery after open fracture. Hispanic patients had slightly lower rates of surgery for both open and closed fractures; however, the differences were not statistically significant. There were no notable insurance-based differences in treatment for closed or open fractures.

**Table 3:** Effects of Patient Demographic and Insurance Data on Surgical Treatment of Forearm Fractures—State Databases

	Relative Risk	95% Confidence Interval	Relative Risk	95% Confidence Interval
	Closed Forearm Fracture		Open Forearm Fracture	
Teen (age 13-18 years)	3.08	2.77, 3.41^a^	1.89	1.62, 2.20^a^
Female sex	0.90	0.84, 0.98^a^	0.94	0.79, 1.11
Race—White	1.00		1.00	
Race—Black	0.57	0.48, 0.66^a^	0.65	0.53, 0.79^a^
Ethnicity—Hispanic	0.88	0.74, 1.03	0.90	0.74, 1.08
Race—Other/Unknown	1.34	1.06, 1.70^a^	0.81	0.62, 1.06
Medicaid as primary payer	0.94	0.82, 1.09	0.89	0.76, 1.05

a Statistically significant difference, *P* < 0.05.

#### National Database

An additional 724,305 closed and 10,035 open fractures were identified from the NEDS database, and 1.1% of closed and 43.6% of open fractures were treated surgically. Using the NEDS national weighted frequencies to estimate the total national prevalence, this amounts to 3,312,807 closed and 46,569 open forearm fractures in the United States, resulting in an estimated 59,024 forearm fractures initially treated with surgery for this period (Table 4). Multivariable regression revealed that teenage patients had increased odds of surgery for closed and open fractures (odds ratio [OR] = 4.205, CI = 3.964, 4.46; OR = 2.172, CI = 1.971, 2.393, respectively) while female patients and patients with Medicaid had lower odds of surgical management for closed fractures (OR = 0.939, CI: 0.888, 0.993; OR: 0.871, CI: 0.821, 0.924, respectively [Table 5]).

**Table 4:** Comparison of National Emergency Department Sample (NEDS) True Records and Estimated Weighted National Frequencies

	True Records		Weighted National Estimate	
	N (%)			
Fracture Diagnosis	Nonsurgical	Surgical	Nonsurgical	Surgical
Forearm—Closed	716,054 (98.9)	8251 (1.1)	3,274,313 (98.8)	38,494 (1.2)
Forearm—Open	5662 (56.4)	4373 (43.6)	26,039 (55.9)	20,530 (44.1)
Forearm—Total	721,716 (98.3)	12,624 (1.7)	3,300,352 (98.2)	59,024 (1.8)
Tibia—Closed	215,686 (96.5)	7838 (3.5)	683,208 (95.0)	36,166 (5.0)
Tibia—Open	2486 (40.0)	3729 (60.1)	9804 (37.5)	16,340 (62.5)
Tibia—Total	218,172 (95.0)	11,567 (5.0)	693,012 (93.0)	52,506 (7.0)

**Table 5:** Effects of Patient Demographic and Insurance Data on Surgical Treatment of Forearm Fractures—NEDS

	Odds Ratio	95% Confidence Interval	Odds Ratio	95% Confidence Interval
	Closed Forearm Fracture		Open Forearm Fracture	
Teen (age 13-18 years)	4.21	3.96, 4.46^a^	2.17	1.97, 2.39^a^
Female sex	0.94	0.89, 0.99^a^	1.00	0.90, 1.11
Medicaid as primary payer	0.87	0.82, 0.92^a^	0.92	0.84, 1.02

a Statistically significant difference, *P* < 0.05

### Tibial fractures

#### State Databases

Fifty thousand six hundred forty patient records with closed and 1339 with open tibial fractures were identified; surgical treatment was done in 7.9% and 60.5% of the cases, respectively.

Multivariable regression showed a slightly increased risk of surgery for the Black and other/unknown race groups (RR = 1.18, CI: 1.02, 1.36, RR = 1.21, CI: 1.04, 1.42, respectively) for closed fractures compared with Whites; no differences were observed for open fractures. Hispanic and White patients had similar surgical rates for closed and open fractures. Based on insurance type, there were no significant differences in the treatment of closed fractures; however, patients with Medicaid were 17% less likely undergo surgery for open fractures (Table 6).

**Table 6:** Effects of Patient Demographic and Insurance Data on Surgical Treatment of Tibia Fractures—State Databases

	Relative Risk	95% Confidence Interval	Relative Risk	95% Confidence Interval
	Closed Tibial fracture		Open Tibial fracture	
Teen (age 13-18 years)	3.55	3.24, 3.88^a^	1.16	1.07, 1.27^a^
Female sex	0.91	0.85, 0.98^a^	0.93	0.84, 1.03
Race—White	1.00		1.00	
Race—Black	1.25	1.06, 1.47^a^	1.01	0.91, 1.11
Ethnicity—Hispanic	1.01	0.85, 1.21	1.01	0.89, 1.15
Race—other/unknown	1.30	1.11, 1.53^a^	1.05	0.93, 1.19
Medicaid as primary payer	0.89	0.81, 0.98^a^	0.83	0.74, 0.93^a^

a Statistically significant difference, *P* < 0.05.

#### National Database

Another 223,524 closed and 6215 open fractures were included from the NEDS. Surgery was the index treatment in 3.5% of closed and 60.0% of open fractures. The weighted national estimate for the study period was 745,518 tibial fractures (719,374 closed, 26,144 open), resulting in 52,506 index surgeries (Table 4). Multivariable regression demonstrated that teens were more likely to receive surgical treatment for closed fractures while female patients were 27% less likely. In addition, Medicaid patients were 20% less likely to have surgery for closed and 23% less likely for open fractures (Table 7).

**Table 7:** Effects of Patient Demographic and Insurance Data on Surgical Treatment of Tibia Fractures—Nationwide Emergency Department Sample

	Odds Ratio	95% Confidence Interval	Odds Ratio	95% Confidence Interval
	Closed Tibial fracture		Open Tibial fracture	
Teen (age 13-18 years)	2.92	2.73, 3.13^a^	1.06	0.92, 1.21
Female sex	0.73	0.69, 0.77^a^	0.95	0.83, 1.09
Medicaid as primary payer	0.80	0.75, 0.86^a^	0.77	0.67, 0.88^a^

a Statistically significant difference, *P* < 0.05.

## Discussion

We report the largest series of pediatric forearm and tibial fractures and have identified several disparities in treatment. We chose to include two of the most common pediatric fractures, forearm and tibia, both because of their incidence and because of the clinical decision making necessary to achieve optimal outcomes given the various methods of treatment and evolving trends of surgical and nonsurgical management.^31^ Although available treatment options have advantages and disadvantages, no evidence exists to support that race/ethnicity or insurance status correlate with any notable physiologic differences affecting healing or specific treatment considerations that would lead to alterations in outcomes based on these factors. Therefore, treatment methods should be similar among all races/ethnicities and payer types. However, we found that Black patients were 43% less likely to receive surgery after closed forearm fractures and 35% less likely after open forearm fractures compared with White patients while Medicaid patients were 17% to 20% and 23% less likely to receive surgery after closed and open tibial fractures, respectively. We did not find any notable differences in the surgical treatment of Hispanic patients compared with Whites for any of the fractures. Importantly, there were no notable differences in surgery rates between state databases, reflecting a lack of state-specific policy or geographic biases in the treatment received. Teenagers were more often treated with surgery, which is expected and likely represents treatment preference, because patients are more likely to require surgery as they reach skeletal maturity. Although we have proven that treatment differences exist among these populations, the significance of these is not clear, especially because many of these fractures are successfully treated without surgery.

Previous studies have attempted to report evidence of ethno-racial and socioeconomic disparities among pediatric long bone fracture patients. However, we think these disparities are both more common than previously reported and have not markedly changed despite interventions to close the gap of inequality. Slover et al. conducted a retrospective review of the HCUP Kid's Inpatient Database for closed supracondylar humerus, femoral shaft, and forearm fractures, demonstrating a notable difference in the treatment of supracondylar humerus fractures based on race/ethnicity, with minorities (Black and Hispanic) more likely to receive closed reduction with percutaneous pinning than White patients.^13^ They also found notable differences in the treatment of femoral shaft fractures based on insurance type, with privately insured children more likely to be treated with an external fixation device than Medicaid and self-pay groups. Interestingly, they found no differences for race/ethnicity or payer type for forearm fractures, which highlights the need for additional evaluation of these issues with investigations such as ours. By contrast, our results demonstrated markedly lower rates of surgery in Black patients with closed and open forearm fractures. Limitations of their study include analysis of only closed fractures, use of a single inpatient database, relatively small population, and narrow age range not encompassing all pediatric patients. Using an inpatient-only database is not well suited for evaluating treatment of pediatric forearm fractures, especially because most are treated in an outpatient setting (only 4.6% from SIDD in our study). This supports the value of the multiple, large databases, including inpatient, emergency department, and ambulatory visits, that we used to increase the population, diversity, and geographic extent to more accurately reflect statewide and national trends while reducing the likelihood of false-negative results. In addition, we examined data from 2006 to 2015, whereas they assessed a single year (2000). Given the time difference in these studies and efforts to address healthcare disparities, these differences could result from the limitations discussed or may indicate that disparities are becoming a more common problem and/or attempted interventions are not providing a notable benefit in reducing them.

The negative clinical,^1,2,6,8,15,16,20,22,32^ fiscal,^17-25-26^ and patient-related significance of ethno-racial and socioeconomic barriers to receiving high-value, high-quality health care have been reported across all medical specialties, including orthopaedic surgery and pediatric orthopaedics. Clinically significant effects in pediatric fracture care include variability in pain management,^21^ timely treatment and access to follow-up care,^8,11,12,20,21,23^ repeat ED visits,^4^ evaluation, and management.^7,8,22^ The significance of these on clinical outcomes is being researched with varying results,^2,15,22^ although there are limited data in the pediatric population. Additional investigation into the effect on outcomes in pediatric fracture patients is necessary to better understand the implications. The economic effect of all race-based healthcare disparities is estimated to be greater than $350 billion annually in the United States, including over $35 billion for excess direct healthcare costs and >$200 billion for indirect costs from disease-related loss of productivity and premature death.^17,25,26^ Limited access to equal, high-quality care may result in delayed diagnosis, treatment, or follow-up, resulting in excess charges (direct) or missed time in the work force/lost productivity (indirect) from preventable complications and illnesses. Therefore, in addition to ethical and moral considerations, there is high incentive for medical providers, administrators, and policymakers to eliminate healthcare disparities.

Our data demonstrated differences in the surgical treatment of closed and open tibial fractures for Medicaid patients compared with other payer types. Furthermore, although we did not find notable differences in treatment based on race/ethnicity for tibial fractures, it has been reported that minorities in the pediatric population are less likely to have insurance or access to regular sources of care.^20^ Because uninsured patients are not captured in these databases, there may be some selection bias intrinsic to these data. Discrepancies in treatment based on insurance status require additional investigation, especially in the setting of Medicaid expansion and a shift toward value-based payment. The evolution of these programs may inadvertently increase disparities and disproportionately affect those it was meant to help through increasing access to insurance and care by placing additional stress on the current healthcare system because of unidentified factors.^3,36,37,38,39,40,41^ In addition, although the percentage of the pediatric population with insurance is increasing, disparities still exist, and access to follow-up orthopaedic care can be difficult, regardless of the insurance type.^4,5,10,11,12,13,14^ It is exceedingly important to continue dedicated research of local, regional, and national trends in healthcare disparities, their causes, and implementation of effective systems to mitigate additional damage caused by these issues. Potential targets for improvement include addressing system-based limitations, dedicating resources to process improvement and multidisciplinary treatment teams, paying for performance and/or change in Medicaid reimbursement practices to incentivize providers and improve access to care, and constant evaluation of policies and practices to determine their effect.^3,16,36,37,38,39,40,41^

Our patient selection and the use of several state and national inpatient, emergency department, and outpatient databases provide greater statistical power and generalizability and more accurate estimates of trends in health care. Our study, however, has limitations intrinsic to administrative data, including accurate reporting and data entry. The effect of this on our findings is unclear. Research to validate the use of administrative data to identify hip fractures in adult patients reported a sensitivity of 67% to 97%.^35^ Another study demonstrated 76% sensitivity and specificity for identifying complications of hip fractures, yet another reported 29% to 100% sensitivity and >92% specificity for complications of total knee arthroplasty.^32,34^ Although high sensitivity allows for the capture of more true cases, false-negative rates may be inflated leading to an overestimation of cases, whereas high specificity would likely underestimate them. Regarding data collection, race and ethnicity were not collected separately, limiting accurate classification of multiracial and multiethnic patients. Data collection including race and Hispanic and other ethnicity details are more commonly used by non-government and government organizations, including the Agency for Healthcare Research and Quality, which will help more accurately characterize populations and disparities in future series. Another limitation is the lack of patient-specific data, which makes detailed analysis of individual cases and indications for particular treatment methods impossible. Inclusion of all patients with a diagnosis, rather than limiting our data by introducing restrictive parameters, allows us to more accurately assess the breadth of treatment for these fractures, providing a more representative sample. In addition, because state and national databases provide data for different populations, patient and insurance characteristics within each state may not be nationally representative, which may account for the differences in surgical rates for Medicaid patients between state and national databases, and is the impetus for including both in our study.

## Conclusion

Ethno-racial and insurance-based disparities persist as barriers to high-value, high-quality pediatric fracture care despite quality improvement efforts. Administrative databases may help identify population-level disparities and monitor progress. Additional investigation and measured interventions to address factors related to inequality are necessary and medical providers, leaders, and policymakers should work together to further define potential targets for improvement.

## Supplementary Material
