# Supplementary material for: Assessment of Ethno-racial and Insurance-based Disparities in Pediatric Forearm and Tibial Fracture Care in the United States
Source: J Am Acad Orthop Surg Glob Res Rev. 2022 Jul 29;6(7):e22.00126. doi: 10.5435/JAAOSGlobal-D-22-00126 (PMC10566840; doi:10.5435/JAAOSGlobal-D-22-00126)
Supplement: Supplementary file 1 [file jagrr-6-e22.00126-s001.docx]

Appendix: ICD-9-CM and CM/CPT-4 Codes used to identify fracture diagnoses and treatments.

| **Diagnosis Codes** | **ICD-9-CM** | |
| --- | --- | --- |
| Forearm Fracture | 813.20-813.23, 813.40-813.47, 813.80-813.83 | |
| Open Forearm Fracture | 813.10-813.18, 813.30-813.33, 813.50-813.54, 813.90-813.93 | |
| Closed Tibia Fracture | 823.00, 823.02, 823.20, 823.22, 823.40, 823.42, 823.80, 823.82, 824.0, 824.8 | |
| Open Tibia Fracture | 823.10, 823.12, 823.30, 823.32, 823.90, 823.92, 824.1, 824.9 | |
| **Procedure Codes** | **ICD-9-CM** | **CM/CPT-4** |
| Surgical Treatment –  Forearm Fracture | 79.22, 79.32, 79.52, 79.92 | 24586, 24587, 24635, 24665, 24666, 24685, 25240, 25400, 25415, 25515, 25525, 25526, 25545, 25574, 25575, 25607, 25608, 25609, 25651, 25652, 25671 |
| Surgical Treatment –  Tibia Fracture | 79.26, 79.36, 79.46, 79.56, 79.66, 79.96 | 27535, 27536, 27756, 27758, 27759, 27766, 27769, 27814, 27822, 27823, 27826, 27827, 27828 |
